# Research on Unsupervised Low-Light Railway Fastener Image Enhancement Method Based on Contrastive Learning GAN

**DOI:** 10.3390/s24123794

**Published:** 2024-06-11

**Authors:** Yijie Cai, Xuehai Liu, Huoxing Li, Fei Lu, Xinghua Gu, Kang Qin

**Affiliations:** 1 China Railway Wuhan Bureau Group Co., Ltd., Wuhan 430061, China; yijie.cai@hbut.edu.cn; 2 School of Mechanical Science and Engineering, Huazhong University of Science and Technology, Wuhan 430074, China; 3 Gemac Engineering Machinery Co., Ltd., Xiangyang 441000, China; lufei0037@163.com (F.L.); guxh123@126.com (X.G.); 15997418455@163.com (K.Q.); 4 School of Mechanical Engineering, Hubei University of Technology, Wuhan 430068, China; 102110165@hbut.edu.cn; 5 Key Laboratory of Modern Manufacturing Quality Engineering in Hubei Province, Wuhan 430068, China

**Keywords:** railway fastener, low-light image enhancement, detection equipment, CES-GAN

## Abstract

The railway fastener, as a crucial component of railway tracks, directly influences the safety and stability of a railway system. However, in practical operation, fasteners are often in low-light conditions, such as at nighttime or within tunnels, posing significant challenges to defect detection equipment and limiting its effectiveness in real-world scenarios. To address this issue, this study proposes an unsupervised low-light image enhancement algorithm, CES-GAN, which achieves the model’s generalization and adaptability under different environmental conditions. The CES-GAN network architecture adopts a U-Net model with five layers of downsampling and upsampling structures as the generator, incorporating both global and local discriminators to help the generator to preserve image details and textures during the reconstruction process, thus enhancing the realism and intricacy of the enhanced images. The combination of the feature-consistency loss, contrastive learning loss, and illumination loss functions in the generator structure, along with the discriminator loss function in the discriminator structure, collectively promotes the clarity, realism, and illumination consistency of the images, thereby improving the quality and usability of low-light images. Through the CES-GAN algorithm, this study provides reliable visual support for railway construction sites and ensures the stable operation and accurate operation of fastener identification equipment in complex environments.

## 1. Introduction

In the railway system, railway fasteners are crucial components—the safety and integrity of which are essential for the safe operation of trains [1]. Issues such as the aging, damage, or loosening of fasteners can threaten the safety and stability of the railway transportation system [2]. Therefore, efficient and accurate fastener defect detection equipment is in need for the safe maintenance and operation of the railway system. However, in actual railway maintenance operations, especially at nighttime, low-light conditions often pose significant challenges for machine vision-based fastener defect detection equipment. The low-light environment not only limits the detection effectiveness of the equipment at nighttime but also degrades the quality of the acquired images, thereby affecting the accurate capture and recognition of defect features.

Low-light image enhancement has become a prominent research topic in the field of computer vision, but studies focusing on the railway fastener domain are still lacking. Introducing low-light image enhancement technology into railway fastener detection is expected to further improve the performance of defect detection equipment under low-light or insufficient lighting conditions, thereby enhancing nighttime operation capabilities. Currently, low-light image enhancement algorithms can be divided into traditional algorithms and deep learning-based algorithms.

Traditional low-light image enhancement algorithms mainly rely on image processing and computer vision techniques, using methods such as brightness compensation [3] and contrast enhancement [4] to improve the quality of low-light images. Examples include the Retinex model [5,6,7,8,9], histogram equalization [10], and grayscale transformation [11]. These methods have obtained some achievements in enhancing image details and contrast, but they still suffer from issues such as noise, artifacts, and loss of details under low-light conditions.

To address these problems, Zhang et al. [12] proposed an underwater image enhancement method based on minimal color distortion and local adaptive contrast enhancement. By combining minimal color distortion and local adaptive contrast enhancement, they effectively improved the visual quality of low-light images. Similarly, Yasaswini et al. [13] proposed a method that achieved good results in the field of underwater image enhancement. Priya et al. [14] further optimized this method, validating its broad applicability and stable performance in different environments. These studies demonstrate that minimal color distortion and local adaptive contrast enhancement can effectively improve image contrast and detail representation under low-light conditions.

However, traditional low-light image enhancement algorithms still have some limitations, such as noise and color distortion issues. Wang et al. [15] proposed an image enhancement method based on weighted wavelet visual perception fusion. By using weighted wavelet transform and visual perception models, they improved image contrast and detail representation, but the method still faced challenges when dealing with high noise and complex lighting conditions.

With the advancement of deep-learning technology, low-light image enhancement algorithms have made significant breakthroughs. Some algorithms utilize convolutional neural networks (CNN) or generative adversarial networks (GAN) to learn feature representations and mapping relationships from large amounts of low-light image data, thereby achieving effective enhancement of low-light images. For example, Guo et al. [16] proposed a low-light image enhancement method based on illumination map estimation, which enhances the effect by estimating the illumination map of low-light images and performs well under complex lighting conditions.

To better handle the details and noise of low-light images, Feng et al. [17] proposed a layered guided low-light image enhancement network. Using dual-tree complex wavelet transform (DT-CWT) for brightness guidance and multiple branches for weak light enhancement tasks, this method can generate more realistic low-light images and improve image detail and clarity.

Additionally, Yang et al. [18] combined adaptive gamma correction with the Retinex model and proposed a low-light image enhancement method based on Retinex decomposition. By computing adaptive gamma correction to adjust image contrast and refining the decomposed illumination map, the method improves the quality of low-light images. Shi et al. [19] proposed a dual super-resolution framework based on deep learning, which enhances the details and clarity of low-light images using a low-light image convolutional neural network and a super-resolution network.

In addressing the low-light image enhancement task for railway fasteners, it is essential to balance noise reduction and detail recovery, color distortion, uneven local lighting, low perceived image quality, and the lack of real paired datasets. This study focuses on a deep convolutional neural network-based low-light image enhancement algorithm, incorporating unsupervised learning and other techniques to deeply explore the potential value of low-light images and provide technical support for the in-depth analysis of low-light images of railway fasteners. This paper proposes a novel unsupervised image enhancement method, CES-GAN, based on EnlightenGAN. Unlike supervised learning, unsupervised learning methods do not require paired training data, thus overcoming data collection difficulties and enabling broader application to different types of image data. Additionally, a feature consistency loss function is introduced to ensure that the content information of the enhanced image is retained, and an illumination color loss is introduced to control the intensity and uniformity of the generated image’s illumination. Furthermore, this paper utilizes contrastive learning to learn feature representations, allowing the model to acquire a better performance of learning image features under various low-light conditions and improving the model’s generalization ability.

The contributions of this paper are as follows:

(1) A contrastive learning framework is introduced. Traditional low-light image enhancement methods often fail to deeply understand the image feature distribution of railway fasteners under low-light conditions. In contrast, the proposed CES-GAN, by utilizing a contrastive learning framework, can more comprehensively understand image features, thereby improving the quality and realism of the generated images. For example, compared to the method by Zhang et al. [12], the CES-GAN can more accurately capture the key features in railway fastener images within the contrastive learning framework, further enhancing image enhancement effects.
(2) A semantic feature loss module is introduced. Previous methods often result in blurred content in railway fastener images and a lack of clear preservation of image semantics. The semantic feature loss module introduced in this paper helps maintain semantic consistency, making the generated railway fastener images clearer and more natural. For instance, compared to the method by Priya et al. [14], the semantic feature loss module in this paper can significantly preserve the semantic information of railway fastener images, improving image clarity and realism.
(3) Traditional methods often fail to effectively handle the problem of inconsistent lighting conditions in low-light railway fastener images. This paper introduces an illumination loss function to constrain the illumination distribution of the generated images, making railway fastener images appear more natural and consistent under various lighting conditions. For example, compared to the method by Feng et al. [17], the illumination loss function in this paper more effectively adjusts the illumination distribution of the images, improving their naturalness and consistency.

## 2. Related Work

Multiple Exposure Fusion Enhancement Method: Multi-exposure fusion enhancement methods improve the quality of low-light images by combining multiple images with different exposures. Akbulut et al. [20] proposed a convolutional neural network-based multi-exposure image fusion method using a deep-learning model. By jointly learning the tasks of low-light image enhancement and multi-exposure fusion, this method achieves better multi-exposure fusion results while enhancing low-light images. Additionally, Jin et al. [21] utilized deep-learning techniques for multi-exposure fusion. By jointly learning the representation and fusion processes of multi-exposure images, their method enhances low-light images while achieving better dynamic range and detail enhancement. These methods offer significant advantages in improving image dynamic range and detail representation, although there is still room for improvement in handling complex lighting conditions.

Global and Local Information Recovery Methods: Global and local information recovery methods enhance low-light images by combining global and local information. Zhu et al. [22] proposed a deep-learning method that integrates illumination map estimation and local context enhancement. By estimating the illumination map and considering local context information, their method enhances the details and contrast of low-light images. To further improve the quality of low-light images, Fu et al. [23] introduced a dual attention network. This network uses attention mechanisms to automatically focus on important details in the image, maintaining naturalness and realism during enhancement. Additionally, Zhang et al. [24] applied joint learning of illumination and texture representations to low-light image enhancement, improving image details, contrast, and texture. To comprehensively utilize global and local information, Zhang et al. [25] enhanced low-light images by jointly learning global and local information representations, thus improving image quality and details. Moreover, to adapt to low-light images from different domains, Zhang et al. [26] employed an adaptive method based on multi-domain learning. By learning low-light image representations from multiple domains, this method better adapts to various low-light conditions and achieves effective image enhancement. Subsequently, Arora A [27] combined global and local contrast enhancement methods to improve the quality of low-light images. By enhancing global contrast and preserving local details, this approach improves the brightness and details of low-light images. These methods, by combining global and local information, comprehensively enhance the quality of low-light images.

Illuminance Curve Estimation Contrast Enhancement Methods: Illumination curve estimation contrast enhancement methods improve image contrast and detail by accurately estimating the illumination curve of the image. Liu et al. [28] combined content and illumination estimation in a deep-learning approach to enhance low-light images. By learning the content and illumination distribution of images, this method improves the details, contrast, and clarity of low-light images. Subsequently, Li et al. [29] employed an adaptive weighted Retinex model to enhance low-light images. By adaptively adjusting the weights of the Retinex model, they improved the brightness, contrast, and detail of low-light images. These methods effectively enhance image contrast and detail representation by accurately estimating illumination curves.

## 3. Methods and Recommendations

This paper proposes an unsupervised low-light image enhancement network called CES-GAN, which achieves high-quality enhancement of low-light images of railway fasteners by designing a unique network structure and introducing multiple loss function modules. The CES-GAN network consists of a generator, a global discriminator, a local discriminator, semantic feature loss, and illumination loss modules. These modules collaborate to effectively extract and reconstruct the details and textures of low-light images while improving the consistency and uniformity of the images’ illumination. Through training on different data-enhanced images, the CES-GAN network demonstrates a good generalization ability under various environmental conditions, providing an innovative solution to the low-light image enhancement problem of railway fasteners.

(1) CES-GAN utilizes a semantic feature loss module to address the issue of blurry image content. Under low-light conditions, images often lack clear textures and details. The semantic feature loss module maintains the semantic consistency of images, making the generated images visually clearer and more natural. By reconstructing the details and textures of the images, CES-GAN provides richer and more realistic image enhancement effects.
(2) CES-GAN introduces a contrastive learning framework. By comparing the model’s input original low-light images with the images after data enhancement, contrastive learning ensures the outputs of the two are as consistent as possible. This allows CES-GAN to learn image features under different low-light conditions, thereby improving the quality and realism of the generated images. The contrastive learning framework helps the network to better understand the distribution of image features under low-light conditions, resulting in the more accurate restoration of image details and textures.
(3) CES-GAN introduces illumination loss to address the issue of inconsistent illumination under low-light conditions. Due to lighting constraints, low-light images often exhibit color distortion and uneven lighting. The illumination loss function constrains the illumination distribution of generated images, making them appear more natural and consistent under various lighting conditions.

### 3.1. Overall Network Architecture

**Figure 1:**
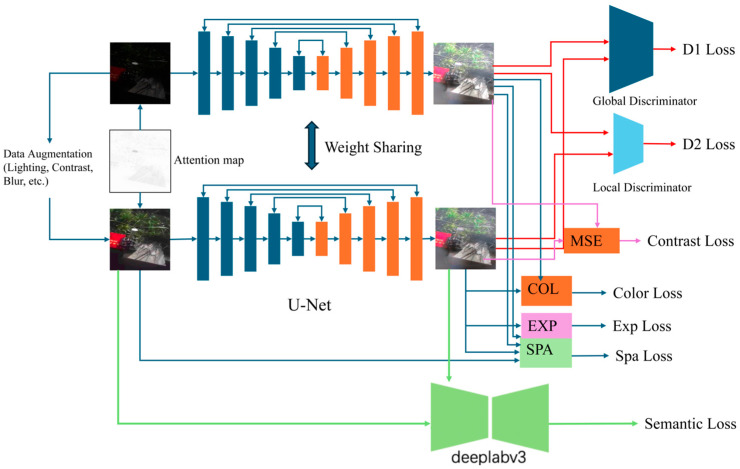
Illustration of the architecture of CES-GAN for low-light image enhancement.

The paper proposes an unsupervised low-light image enhancement network called CES-GAN. Firstly, the CES-GAN is a method that does not require paired training datasets for model training. By introducing contrastive learning frameworks and other solutions, it achieves the model’s generalization and adaptability under different environmental conditions, where the red book in the input image represents a substitute for the track in data capture sampling. The network structure of CES-GAN is illustrated in Figure 1. Secondly, its generator adopts a U-Net model with five layers of downsampling and upsampling structures, effectively extracting and reconstructing image details and textures, thereby improving issues such as low contrast and blurriness in low-light images. This enables CES-GAN to generate more realistic and clearer image enhancement results. To further enhance the realism and detail of the enhanced images, CES-GAN introduces two types of discriminators: global and local discriminators, which focus on overall and local features, respectively. This helps the generator to preserve image details and textures during the reconstruction process, improving the details and textures of the enhanced images. Discriminator losses (D1 loss and D2 loss) are used to measure the differences between generated images and real images. To maintain semantic and spatial consistency between generated and input images, CES-GAN introduces semantic feature consistency loss (semantic loss) and spatial feature consistency loss (spatial loss). Additionally, to improve the lighting and color consistency of generated images, CES-GAN introduces exposure control loss (exp loss) and color constancy loss (color loss). Finally, by introducing a contrastive learning framework and training on input images enhanced with different data-augmentation techniques, the contrastive learning loss (contrast loss) enhances the model’s ability to generalize under different environmental conditions.

### 3.2. Generator Structure

The U-Net architecture is frequently employed in tasks such as semantic segmentation, image restoration, and image enhancement, demonstrating an excellent performance. It comprises an encoder, a middle layer, and a decoder. The encoder consists of multiple downsampling layers designed to progressively reduce the image size and extract features. Each downsampling layer typically consists of two consecutive convolutional layers followed by a pooling layer (often max-pooling or strided convolution). This architecture gradually reduces the image size while capturing features at different scales. The middle layer, between the encoder and decoder, is a convolutional layer. It connects the encoder and decoder, aiding in retaining richer feature information. The decoder section comprises multiple upsampling layers used to progressively increase the image size and perform feature reconstruction. Each upsampling layer typically consists of a transposed convolutional layer (also known as deconvolution) followed by a convolutional layer. Through these layers, the image size is gradually increased, and features are reconstructed. Crucially, skip connections are employed to concatenate features from the encoder to their corresponding features in the decoder. Skip connections help to combine low-level and high-level features, enhancing the model’s ability to capture feature details. In these skip connections, features from the encoder and decoder are concatenated (or stitched together) to form feature fusion. This approach facilitates the fusion of features at different scales, providing richer information to the decoder.

**Figure 2:**
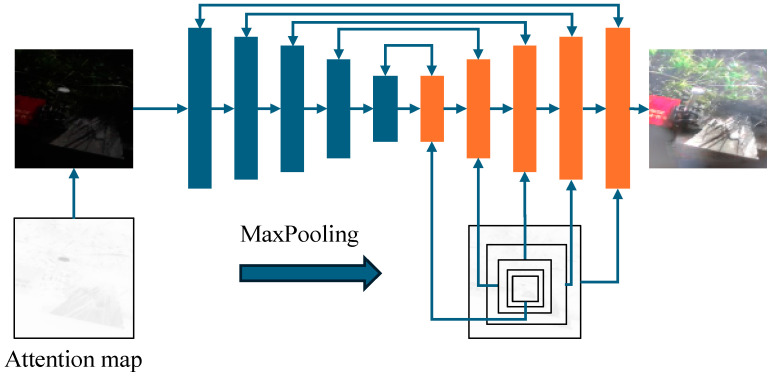
Generator structure.

In this study, an attention-guided U-Net is employed as the generator, and its network structure is illustrated in Figure 2. The attention map of the low-light image is simultaneously inputted along with the image, aiming to focus the model on the contextual information within the scene image. The input image size for the generator is 256 × 256 × 3, and the images are subjected to normalization, performed independently on each channel (such as the RGB channels). Normalization aids in centralizing the data distribution and rendering it to similar scales, thereby facilitating the training of the model. Subsequently, attention maps are computed using the normalized images, as depicted by Equation (1).

(1) $$Attention\_map=\frac{1-(x\_r\times a+x\_g\times b+x\_b\times c)}{2}$$

Here, *x* represents the input normalized image, while *x_r*, *x_g*, and *x_b* denote the features of the red, green, and blue channels of the image, respectively. The parameters *a*, *b*, and *c* are the weights for the red, green, and blue channels, respectively. The coefficients *a*, *b*, and *c* are optimized during the training process to ultimately obtain the optimal weights for generating the attention map. Incorporating the attention map in the first layer can lead to the loss of attention information in subsequent features. Therefore, the maxpooling operation is applied to the attention map to obtain multi-scale feature attention. During each upsampling process, the attention is multiplied by the current feature map to ensure that the attention information is propagated throughout the entire network.

The normalized image and the obtained attention are concatenated along the channel dimension to form a new feature with attention. The input features undergo downsampling operations using the U-Net encoder, where the convolutional kernel size is 3 × 3, with a stride of 1 and padding of 1, and the pooling layer is max-pooling with a kernel size of 2 × 2 and a stride of 2. Then, they pass through the middle layer, where the convolutional kernel size is 3 × 3, with a stride of 1 and padding of 1. The deconvolutional kernel size in the upsampling layers of the decoder is 2 × 2, with a stride of 2, and the convolutional kernel size is 3 × 3, with a stride of 1 and padding of 1. After each convolution, ReLU function and batch normalization are applied, and after every 2 convolutions, max-pooling is applied again.

### 3.3. Discriminator Structure

The discriminator in this study consists of a global discriminator and a local discriminator, employing adversarial learning methods to evaluate the authenticity of images generated by the generator. During the training process, the generator generates samples, which are then passed to the discriminator. Subsequently, the discriminator classifies these samples based on their authenticity. The objective of the generator is to render the discriminator incapable of accurately distinguishing between generated samples and real samples, thereby generating realistic samples. Conversely, the objective of the discriminator is to accurately distinguish between real samples and generated samples as much as possible.

**Figure 3:**
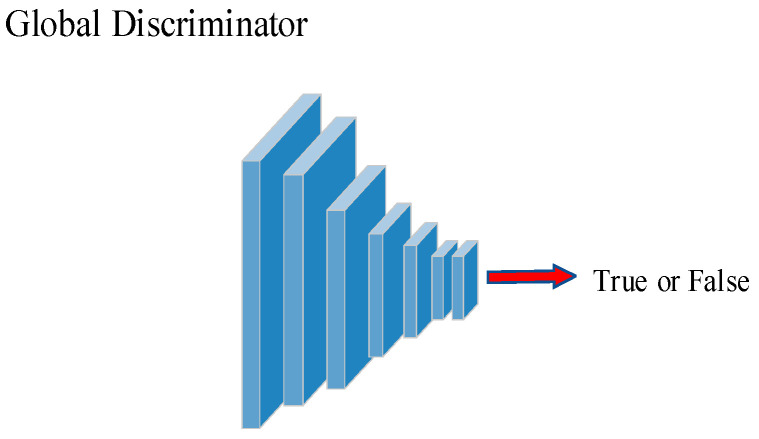
Structure of the global discriminator.

**Figure 4:**
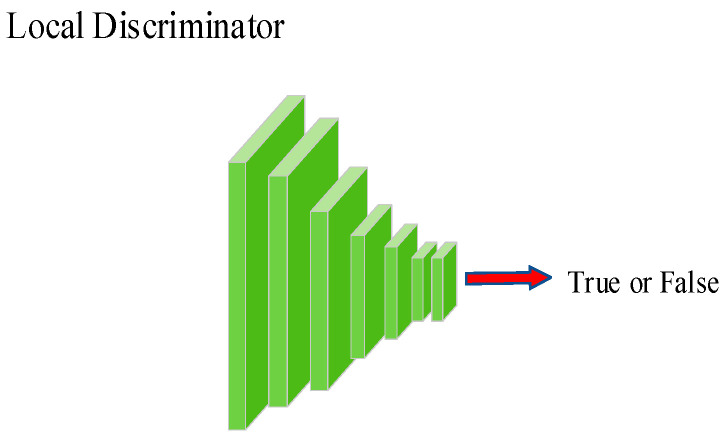
Structure of the local discriminator.

The structure of the discriminator consists of 6 convolutional blocks, with Leaky ReLU activation applied after each convolutional layer. The convolutional kernels used have a size of 4 × 4, a stride of 1, and padding of 1. This architecture effectively extracts features from the images, aiding the discriminator in accurately classifying real and generated samples. The structures of the global discriminator and the local discriminator are depicted in Figure 3 and Figure 4, respectively.

In the railway fastener enhancement stage, the design of the global and local discriminators enables the model to pay more attention to the details of each patch by randomly cropping the output images to a fixed small size and then discriminating the small-sized images. This helps to avoid local areas being too dark or too bright, thereby improving the quality and realism of the enhanced images. The loss function of the discriminator typically employs the adversarial loss function, where the generator aims to produce images that cannot be accurately distinguished from real images by the discriminator, while the discriminator aims to classify real and generated images as accurately as possible. This adversarial training process continually encourages the generator to improve the quality of the generated images, while the discriminator also learns and enhances its classification ability, thus optimizing the model.

The design of the global and local discriminators enables the model to pay more attention to the details of the image, particularly in handling local lighting details in the railway fastener enhancement domain. By cropping the images and employing local discrimination, the discriminator can better assess the authenticity of the images and guide the generator to produce more realistic and detail-rich enhanced images. Ultimately, the railway fastener images generated by the CES-GAN model are effectively enhanced in terms of lighting and details, thus improving the usability and credibility of the images.

### 3.4. Loss Functions

In the CES-GAN network, the loss functions mainly consist of the generator loss function and the discriminator loss function. The generator loss function is a crucial component of the CES-GAN network, responsible for measuring the difference between the generator network’s output images and the real images. By minimizing this difference, it enhances the performance of the generator and the quality of the output images. Simultaneously, the discriminator loss function in the CES-GAN network is used to evaluate the discriminator network’s ability to differentiate between the fake images generated by the generator and the real images. Minimizing this loss function improves the network’s ability to effectively distinguish between real and generated images. The generator loss function and discriminator loss function interact in the CES-GAN network, jointly driving the network’s learning and optimization process. The generator optimizes the generator loss function to generate more realistic images, while the discriminator optimizes the discriminator loss function to improve its ability to discriminate between real and generated images.

#### 3.4.1. Generator Loss Function

In the CES-GAN network, the generator utilizes $L_{G}^{glob}$ and $L_{G}^{loc}$ during the training process. These two loss functions for the generator are aimed at improving the quality and realism of the generated images through adversarial learning. The formula for the generator’s GAN training loss function is shown in Equations (2) and (3).

Firstly, $L_{G}^{glob}$ loss function represents the global adversarial loss, which trains the generator to deceive the global discriminator, making the generated images more realistic overall. The goal of $L_{G}^{glob}$ loss function is to minimize the sum of the logarithm of the probabilities of real images being classified as real and generated images being classified as fake. This forces the generator to produce more realistic images to deceive the discriminator, thereby enhancing the overall quality of the generated images. Secondly, the $L_{G}^{loc}$ loss function represents the local adversarial loss, which improves the local details of the generated images in an adversarial learning manner, making them more realistic. The $L_{G}^{loc}$ loss function achieves this by minimizing the distinguishability of the local discriminator between real and generated images. This helps the generator to focus more on the local details of the images, making the generated images more realistic in local regions. As training progresses, the values of $L_{G}^{glob}$ and $L_{G}^{loc}$ gradually decrease because the quality of the generated images generated by the generator improves and approaches the distribution of real images. When the values of these two loss functions converge to a small stable value, it indicates that the generator has learned the distribution of the data, and the quality of the generated images has reached the expected level. 

Therefore, by using the $L_{G}^{glob}$ and $L_{G}^{loc}$ loss functions, the CES-GAN generator can gradually improve the overall quality and local details of the generated images through adversarial learning, thereby achieving better model training results and enhancing the quality of the generated images.

(2) $$L_{G}^{glob}=E_{x}[\log D_{glob}(x)]+E_{z}[\log(1-D_{glob}(G(x)))]$$

(3) $$L_{G}^{l\mathrm{oc}}=\sum_{i=1}^{N}E_{x}[\log D_{loc}(x)]+E_{z}[\log(1-D_{loc}(G(x)))]$$

The $L_{G}^{glob}$ function represents the global adversarial loss, where *x* denotes real images, *z* denotes random noise, *G*(*x*) is the image generated by the generator, and $D_{\mathrm{glob}}$ is the global discriminator. This loss function trains the generator through adversarial learning to make the generated images more realistic overall. The $E_{x}[\log D_{glob}(x)]$ represents the logarithm of the probability that real images are classified as real, while $E_{z}[\log(1-D_{glob}(G(x)))]$ represents the logarithm of the probability that generated images are classified as fake. The objective of the generator is to minimize this loss, thereby deceiving the global discriminator and making it unable to accurately distinguish between generated images and real images.

The local adversarial loss is trained through adversarial learning to make the generated images more realistic in local regions. *N* represents the number of local blocks into which the image is segmented, and $D_{loc}^{i}$ is the *i*-th local discriminator. Similar to the global adversarial loss, the objective of the generator is to minimize this loss, thereby deceiving the local discriminator and making it unable to accurately distinguish between generated images and real images.

#### 3.4.2. Feature Consistency Loss

(1) Semantic Feature Consistency

Under low-light conditions, images may suffer from unclear details and low contrast. By introducing semantic segmentation, the semantic information in the image can be effectively preserved and emphasized. Semantic segmentation helps to identify and segment different objects and regions in the image, thereby preserving important semantic structures and information in enhancement algorithms. It also helps to constrain the effects of enhancement algorithms, avoiding over-enhancement or producing unnatural results. By restricting enhancement operations to the regions indicated by semantic segmentation results, it ensures that the enhanced image maintains semantic consistency and avoids unnecessary enhancement of the background or irrelevant regions.

Therefore, this paper introduces the DeepLabv3 model as the semantic segmentation model, which is used to classify each pixel in the input image into different categories with high model accuracy and efficiency. The model employs dilated convolutions in its convolutional layers, aiming to increase the receptive field of the convolutional kernels by introducing holes in them, thereby enhancing the model’s ability to recognize large objects. Non-linearity is introduced after each convolutional layer by applying activation functions, enabling the model to extract information from the scene and achieve pixel-level segmentation. The output of the feature extraction part of the DeepLabv3 model, denoted as *F*(*X*), represents the segmentation mask obtained after feature extraction of the input image X by the DeepLabv3 model. Similarly, for the generated image *G*(*X*), its segmentation mask can also be obtained through the DeepLabv3 model, denoted as *F*(*G*(*X*)). The semantic feature consistency loss can be expressed as the distance between the feature maps, with its loss function formula shown in Equation (4).

(4) $$I_{sem}=\left\|F(X)-F(G(X))\right\|$$

where *F* represents the segmentation model, indicating the segmentation mask obtained from the input image *X* through the segmentation network; *F*(*G*(*X*)) represents the segmentation mask obtained from the generated image input to the segmentation network; and ‖ denotes the Euclidean distance between vectors. By minimizing the semantic feature consistency loss, the generated images can maintain semantic consistency with the original images, thereby achieving semantic feature consistency. This formulation using the DeepLabv3 model as the semantic feature consistency loss function effectively guides the generator to learn to preserve the semantic information of the images, thus generating enhanced images with good semantic features.

(2) Spatial feature consistency loss

Spatial feature consistency loss is a loss function used to promote spatial consistency in image enhancement results. Its primary objective is to ensure spatial consistency in enhanced images by preserving the differences in adjacent regions between the input image and its enhanced version. Specifically, this loss function promotes spatial consistency by measuring the differences in adjacent regions between the input image and the enhanced image, thereby preserving the structure and spatial distribution of the image. The formulation of this loss function is shown in Equation (5).

(5) $$L_{spa}=\frac{1}{N}\sum_{i=1}^{N}\sum_{j\in \Omega (i)}(\left|\left(G(X)\_i-G(X)\_j\right)\right|-\left|X\_i-X\_j\right|{)}^{2}$$

wherein *X* represents the input image, *G*(*X*) denotes the enhanced image, N is the number of local regions, and *Ω*(*i*) represents the four neighboring regions (top, bottom, left, right) centered around region *i*.

The reason for employing the spatial feature consistency loss in the generator is that this loss function helps the generator to learn the spatial structure and distribution information between the input and enhanced images, thereby maintaining spatial consistency during the enhancement process. By minimizing the differences between neighboring regions of the input and enhanced images, the generator can preserve the details and structure of the image, avoiding spatial distortions or deformations in the enhancement results.

The spatial feature consistency loss plays a crucial role in the enhancement of railway fastener images. Railway fastener images typically contain rich spatial structures and detail information, which are essential for ensuring the safety and efficiency of railway transportation. By incorporating the spatial feature consistency loss into CES-GAN, the spatial structures and details in railway fastener images can be effectively preserved, thereby enhancing the quality and usability of the enhanced images.

Specifically, the spatial feature consistency loss optimizes the training process of the generator to better preserve the spatial consistency and accuracy of railway fastener images during enhancement. By minimizing the spatial differences between the input and enhanced images, the generator can effectively extract and retain detailed information from the images, resulting in enhanced images that are more structured and recognizable. This ability to maintain spatial consistency contributes to the visualization and analysis of railway fastener images and enhances their effectiveness in subsequent railway transportation monitoring and management applications.

Finally, by introducing spatial feature consistency loss, CES-GAN can effectively preserve the spatial structure and detail information of railway fastener images during the enhancement process, thereby improving the quality and usability of the enhanced results.

#### 3.4.3. Contrastive Learning Loss

Introducing contrastive learning into low-light enhancement tasks can enhance the model’s robustness and generalization ability. Contrastive learning enables the model to learn feature representations by comparing the similarity between different samples, allowing the model to better capture the features of images under low-light conditions, thereby improving image quality and clarity.

The paper first inputs the input image *X* into the generator, generating the high-light image *G*(*X*). Simultaneously, random data-augmentation operations such as changes in lighting, contrast, brightness, and blur are applied to the input image, resulting in the augmented image *X*, which is then input into the generator to generate a second high-light image *X_aug*. Subsequently, the mean squared error (MSE) loss function is employed to compute the loss between the two generated high-light images, facilitating the training process of the generator and aiding the model in learning more data-augmentation strategies, thereby enhancing the model’s generalization capability.

For the railway fastener figure enhancement task, contrastive learning loss plays a crucial role. Enhancing railway fastener images requires preserving key details such as the shape, size, and position of the fasteners, while also improving the brightness and contrast of the image. Through contrastive learning loss, the generator can learn these critical details effectively, ensuring that the generated enhanced images retain the important features of the railway fasteners while also having better visual effects and realism. The formula for the contrastive loss is shown in Equation (6).

(6) $$L_{cont}=\frac{1}{N}\left\|G(X)-G(X\_aug)\right\|$$

where *N* represents the number of samples, *G*(*X*) denotes the generated image, and ‖ represents the Euclidean distance between vectors.

By comparing the mean squared error between *X* and *X_aug*, the generator can better learn the differences between images, thereby improving the quality and realism of the generated images. This usage of contrastive learning loss helps the generator to learn more data-augmentation strategies, thus enhancing the model’s generalization capability and effectively improving the visual quality of the generated images.

#### 3.4.4. Illumination Loss

(1) Exposure control loss (ECL) is a loss function used in generative adversarial networks (GANs) aimed at suppressing areas of insufficient or excessive exposure in images to enhance the overall exposure quality. This loss measures the distance between the average intensity value of local regions and a preset level of good exposure, denoted as *E*. In this paper, *E* is set to the grayscale level in the RGB color space, established at 0.6 based on existing practices. Although setting *E* within the range [0.4, 0.7] in experiments did not significantly affect performance, for the sake of consistency, this paper opted for 0.6. The formula for exposure control loss is shown in Equation (7).

(7) $$L_{\exp}=\frac{1}{N}\sum_{i=1}^{N}{\left\|\mu (X_{i})-E\right\|}^{2}$$

where $X_{i}$ represents the local region of the input image, $\mu (X_{i})$ denotes the average intensity value of this local region, and E represents the preset level of good exposure. The exposure control loss is utilized within the generator to regulate the exposure level of the generated images, ensuring that the exposure quality meets expectations. During the railway fastening enhancement phase, the exposure control loss effectively adjusts the brightness and darkness of images, making the generated images more balanced and reasonable in overall exposure, thereby enhancing the visualization quality of railway fastening images. This adjustment helps to improve the visual perception of images and better meets the requirements of practical application scenarios. Ultimately, through the introduction of the exposure control loss, the CES-GAN model can generate railway fastening images with good exposure, making the images clearer and brighter, while retaining essential detail information.

(2) The purpose of color constancy loss is to correct potential color biases in enhanced images based on the assumption of color constancy in a gray world, where the colors in each sensor channel average to gray across the entire image. This loss function aims to adjust and establish relationships between the three channels after correction of color deviations in the enhanced image. The formula for color constancy loss is represented as Equation (8).

(8) $$L_{col}=\frac{1}{N}\sum_{i=1}^{N}{\left\|\mu \left(R_{i}-G_{i}\right)\right\|}^{2}+{\left\|\mu \left(R_{i}-B_{i}\right)\right\|}^{2}+{\left\|\mu \left(G_{i}-B_{i}\right)\right\|}^{2}$$

where *R_i_*, *G_i_*, and *B_i_* represent the red, green, and blue channels, respectively, in the enhanced image, *μ* denotes the mean value for each channel, and *N* represents the number of pixels in the image.

The color constancy loss is utilized in the generator to correct color deviations in the enhanced image, ensuring that the generated image exhibits a balanced and consistent color distribution overall, aligning with human visual perception. In the railway fastener enhancement stage, the color constancy loss effectively adjusts the color distribution of the image, making the enhanced image appear more natural and realistic, thereby enhancing the credibility and usability of the image.

By introducing color constancy loss, the CES-GAN model can generate railway fastener images with more balanced and consistent colors. Such images closely resemble the color distribution in real scenes, enhancing the recognizability and reliability of railway fastener images. Ultimately, the images generated by the CES-GAN model are not only improved in terms of exposure but also optimized in terms of color, thereby enhancing the overall quality and realism of the images.

The overall generator loss function of the proposed model is presented in Equation (9).

(9) $$L\_G=L_{G}^{glob}+L_{G}^{loc}+L_{sem}+L_{spa}+L_{cont}+L_{\exp}+L_{col}$$

#### 3.4.5. Discriminator Loss Function

The CES-GAN discriminator training simultaneously employs both the discriminator and loss functions, aiming to enhance the model’s ability to distinguish between real and generated images and guide the generator to produce more realistic images.

Firstly, the loss function (adversarial loss of the local discriminator) trains the local discriminator to distinguish between real local regions and generated local regions, thereby encouraging the model to focus on the details of the image and improve the local realism of the generated images. The loss function is formulated as shown in Equation (10).

(10) $$L_{D}^{Loc}=E_{x_{real}^{loc}}[\log D_{loc}(G{\left(x\right)}^{loc})]+E_{x_{fake}^{loc}}[\log(1-D_{loc}(G{\left(x\right)}^{loc}))]$$

where $x_{real}^{loc}$ represents the local region of the real image, $x_{fake}^{loc}$ represents the corresponding local region of the generated image, and $G{\left(x\right)}^{loc}$ represents the locally generated image. $D_{loc}$ is the local discriminator. Through $L_{D}^{loc}$ loss, the discriminator is trained to better distinguish between real local regions and generated local regions, thereby enhancing the local realism of the generated images.

Next, the $L_{D}^{glob}$ loss function (adversarial loss of the global discriminator) trains the global discriminator to distinguish between the overall authenticity of real images and generated images, encouraging the generator to produce more realistic overall images. The $L_{D}^{glob}$ loss function is formulated as shown in Equation (11).

(11) $$L_{D}^{glob}=E_{x_{real}^{glob}}[\log D_{glob}(x_{real}^{glob})]+E_{x_{fake}^{glob}}[\log(1-D_{glob}(x_{fake}^{glob}))]$$

where $x_{real}^{glob}$ represents real images, $x_{fake}^{glob}$ represents generated images, $G{\left(x\right)}^{glob}$ represents global generated images, and $D_{\mathrm{glob}}$ is the global discriminator. Through $L_{D}^{glob}$ loss, the global discriminator is trained to more accurately distinguish the differences between real images and generated images, guiding the generator to produce more realistic overall images.

The changes in the values of these two loss functions guide the discriminator to better distinguish between real and generated images, thereby improving the quality of generated images by the generator. As the model training progresses, if the generated images become closer to real images, the discriminator will find it more difficult to distinguish between real and generated images, resulting in a decrease in the loss function values. Therefore, the decrease in loss function values reflects the improvement in the model’s performance, indicating that the generated images are closer to the real images.

The overall discriminator loss function of the proposed model is shown in Equation (12).

(12) $$L\_D=L_{D}^{loc}+L_{D}^{glob}$$

## 4. Experiment

To verify the low-light image enhancement performance of the proposed unsupervised CES-GAN algorithm model, this paper will conduct training and evaluation on the R-F-datasetv2 railway fastener dataset. Firstly, the basic settings of the experiments, the dataset used, and the image evaluation metrics are introduced. Subsequently, relevant experimental designs and result analyses are conducted to validate the feasibility of the CES-GAN algorithm.

### 4.1. Experimental Setup

In this study, the experimental setup involves utilizing the PyTorch 1.9.0 deep-learning framework to build the CES-GAN algorithm model. The experimental hardware setup and its parameters are outlined in Table 1.

To address the problem of low-light image enhancement, a set of experimental parameters was designed in this study. Firstly, an unpaired dataset mode was employed to better handle the image enhancement task under low-light conditions of railway fastener images.

In the generator section, this study employed the unet_resize architecture. This architecture is suitable for preserving detailed information in images and enhancing the quality of generated images. For the discriminator section, this study opted for the no_norm_4 structure, where the patchD and patch_vgg parameters were set to 1, while patchD_3 was set to 5. Such settings facilitate more accurate differentiation between real and generated images by the network and enable the capture of local detail information.

To balance computational complexity and image resolution, this study set the image size to 320 × 320, with a local region size of 32 × 32. Additionally, self-attention mechanisms were introduced to enable the network to focus more on important areas, thereby enhancing the effectiveness of image enhancement. Regarding feature loss, this study utilized the VGG network, selecting the relu5_1 layer as the feature extractor, which allows for better capture of the semantic information in images. Experiments were conducted on GPU devices to expedite training speed. Furthermore, contrastive learning and illumination loss mechanisms were introduced, along with the application of semantic segmentation networks, to improve the model’s generalization capability and image enhancement effectiveness.

The configuration of the experimental parameters mentioned above contributes to the improvement of low-light image quality. Through the appropriate selection of network architectures and parameter settings, it is possible to effectively preserve image details, enhance brightness and contrast, and improve overall image quality. Such experimental settings are conducive to enhancing the effectiveness of image enhancement under low-light conditions for railway fasteners.

#### 4.1.1. Dataset

In this study, the R-F-datasetv2 railway fastener dataset was selected as the primary data source for low-light image enhancement. This dataset consists of real railway fasteners, making it highly practical and representative, which is well-suited for the unpaired railway fastener enhancement research conducted in this study.

By establishing the R-F-datasetv2 dataset, this study can simulate real railway scenes, thus enhancing the practicality and accuracy of the algorithmic models proposed in this paper.

During the data-collection process, 2000 RGB low-light images and 2000 RGB high-light unpaired images were selected as the training set, while 50 RGB low-light images and 50 RGB high-light unpaired images were chosen as the test set. This selection aims to ensure that the algorithmic models proposed in this paper perform well under various lighting conditions and exhibit strong generalization capabilities. To obtain high-quality images, multiple brands of camera were used for image capture, diversifying the dataset and enhancing its robustness. Additionally, a few natural-light railway fastener images were downloaded from the internet to enrich the dataset, ensuring its comprehensiveness and diversity while increasing the quantity and quality of images, thereby providing more reliable data support for this study.

Overall, by selecting the R-F-datasetv2, employing multiple brands of camera for image capture, and supplementing with natural-light images, this study aimed to create a comprehensive and diverse unpaired dataset, providing a solid foundation and reliable support for research on unsupervised low-light image enhancement of railway fasteners.

#### 4.1.2. Evaluation Metrics

In this study, performance of the algorithm model is comprehensively evaluated using metrics including the peak signal-to-noise ratio (PSNR) [30], structural similarity (SSIM) [31], mean squared error (MSE) [32], learned perceptual image patch similarity (LPIPS) [33], and natural image quality evaluator (NIQE) [34].

Firstly, PSNR is a classical image quality assessment metric, originating from the field of signal processing, primarily used to measure the degree of distortion between the original and enhanced images. Specifically, PSNR evaluates image quality by calculating the mean squared error (MSE) between the pixel values of the images, which is then converted into a value in decibels (dB), reflecting the clarity and fidelity of the images.

Secondly, SSIM is another commonly used image quality assessment metric, which considers the structural information of images and assesses image quality by comparing the structural similarity between the original and enhanced images. The calculation process of SSIM involves comparing the means, variances, and covariances of the images, resulting in a similarity index ranging between −1 and 1.

Furthermore, MSE is a fundamental image quality assessment metric, measuring the degree of distortion in images by calculating the difference between the pixel values of the original and enhanced images. A smaller MSE indicates less difference between the enhanced and original images, indicating a higher image quality.

Simultaneously, LPIPS is a deep learning-based image quality assessment metric that evaluates the perceptual similarity between enhanced and original images by learning perceptual features using neural network models. The calculation formula of LPIPS is complex, mainly involving the extraction of perceptual features from images and calculating the distance between them to assess image quality. A smaller LPIPS value indicates a higher similarity between two images. In low-light image enhancement, LPIPS can comprehensively evaluate the perceptual quality of enhanced images, particularly in generating texture features, aiding in selecting the optimal enhancement method.

Finally, NIQE is a widely used no-reference image quality assessment metric for image reconstruction tasks, based on the natural scene statistics (NSS) model, which constructs a set of “quality-perception” statistical feature sets for quantifying the quality of test images. NIQE extracts NSS features from test images and utilizes multivariate Gaussian models (MVG) to describe the statistical distribution of these features. Then, NIQE evaluates image quality by comparing the distance between the MVG of the evaluated image and the MVG of quality-perception features extracted from a natural-image database. A smaller NIQE value indicates a better perceived image quality, with more natural texture features.

These metrics offer accurate assessments of image perceptual quality, aiding in evaluating the realism and visual comfort of enhanced images.

#### 4.1.3. Ablation Experiments and Analysis

The paper introduces three key modules in the CES-GAN model: semantic segmentation loss, contrastive learning, and illumination loss. The introduction of these modules has a significant impact on the model’s performance. In order to further investigate the contribution of each module to the model’s performance, a series of ablation experiments were conducted, including qualitative and quantitative experiments. The experimental results on the R-F-datasetv2 railway fastener dataset are shown in Table 2, and the experimental results on the LOL dataset are shown in Table 3. In these tables, an upward arrow (↑) indicates an improvement in value compared to the previous module addition, while a downward arrow (↓) indicates a decrease in value. Specifically, higher values of PSNR and SSIM denote better-enhanced image quality, whereas lower values of MSE, LPIPS, and NIQE denote better-enhanced image quality.

Through Table 2 and Table 3, it can be clearly observed that removing any module has an impact on the performance of railway fastener or low-light image enhancement. On the R-F-datasetv2 dataset, when the CES-GAN algorithm lacks lighting consistency, semantic information, and contrastive learning, their NIQE values are 2.981, 2.797, and 2.851, respectively. Compared to the scenario where all modules are present, their NIQE values increase by 0.348, 0.2, and 0.254, respectively. On the LOL dataset, when the CES-GAN algorithm lacks lighting consistency, semantic information, and contrastive learning, their NIQE values are 2.985, 2.864, and 2.902, respectively. Compared to the scenario condition where all modules are present, their NIQE values increase by 0.268, 0.129, and 0.185, respectively. The ablation results further demonstrate the significant impact of each module on the enhancement effect of railway fastener images.

**Figure 5:**
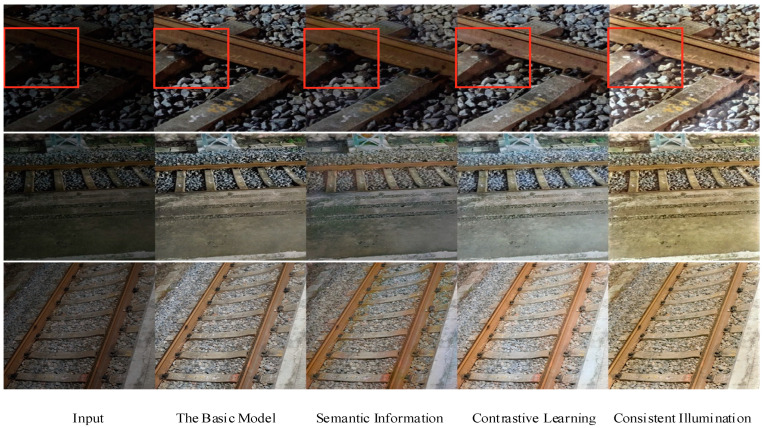
Visualization results of ablation experiment modules.

**Figure 6:**
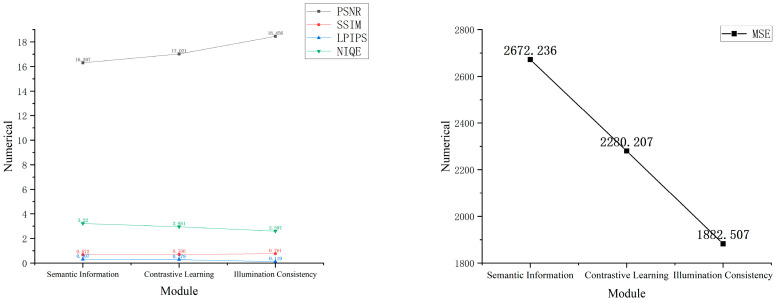
Image parameter metric curves of the CES-GAN algorithm on R-F-datasetv2.

In the qualitative experiments, detailed analyses were conducted on each module, and the model’s intermediate results were visualized to intuitively observe the effects of each module. Start from the baseline model, which served as the starting point of the experiments and was trained without any additional loss functions. In this scenario, the basic performance of the baseline model was observed, indicating how the model performs in railway fastener enhancement without introducing additional modules. Next, semantic consistency loss was introduced. By combining semantic information loss with the baseline model, it was expected that the model could better understand the semantic information of different regions in the image and enhance them accordingly. The introduction of this loss function made the model pay more attention to the features of different parts of the image, thereby improving the enhancement effect. Based on the baseline model and semantic information loss, contrastive learning loss was further introduced. The introduction of contrastive learning aimed to help the model to learn more discriminative feature representations to better distinguish between target objects and backgrounds, thereby further enhancing the enhancement effect. The adaption of this loss function enabled the model to learn richer feature representations, thereby improving the accuracy and stability of railway fastener enhancement. Lastly, lighting loss was introduced and combined with the baseline model, semantic information loss, and contrastive learning loss. The adaption of lighting loss aimed to help the model to better control the exposure level of the image, thereby further enhancing the enhancement effect. The introduction of this loss function enabled the model to handle changes in the image’s lighting conditions, thereby further improving the enhancement results for railway fasteners. The visualization results of the modules and the curves of the image parameter indicators for each module are shown in Figure 5 and Figure 6. In Figure 5, the red box represents a close-up of the details of the railway fasteners, providing additional insights into the enhancement process. The images from left to right represent the input low-light image, baseline results, results with the addition of semantic information loss to the baseline model, results with the adaption of contrastive learning to the baseline model, and results with the incorporation of lighting loss to the baseline model.

From Figure 5, it can be observed that the results generated by the base model, EnlightenGAN, suffer from underexposure and uneven lighting. For instance, in the first row of images, the enhanced result by the base model exhibits significant darkness around the railway fasteners, while other areas appear brighter. This indicates limitations of the base model in handling uneven lighting conditions. Subsequently, upon the introduction of the semantic information loss module, it is observed that the model better understands and extracts semantic information from the images, resulting in enhanced images that are more interpretable and semantically consistent. The addition of the contrastive learning module further improves the clarity of the images, making the generated images sharper and more realistic, particularly in handling subtle variations and detail information. Finally, the introduction of the illumination loss module enables the model to better learn the lighting distribution and variation patterns in the images, thereby enhancing the realism and fidelity of the generated images. With the inclusion of the illumination loss module, the lighting in the railway fastener images generated by the model becomes more uniform, avoiding obvious brightness inconsistencies.

In Figure 6, the evaluation curves after the introduction of each ablation module show continuous improvement in PSNR and SSIM parameter values and a continuous decrease in MSE, LPIPS, and NIQE, further demonstrating the enhancing effects of the modules on railway fasteners.

The synergistic effects of the three modules lead to significant improvements in low-light image enhancement tasks for the CES-GAN model. The semantic information loss module enhances the semantic accuracy of the images, the contrastive learning module improves the clarity of the details in the images, and the illumination loss module enhances the realism of the images. The collaborative action of these modules enables CES-GAN to generate low-light image enhancement results that are of higher quality, more realistic, and more interpretable.

#### 4.1.4. Quantitative Experiment Results and Analysis

In the quantitative experiments, the same training and testing data were used across different models. Performance metrics such as the peak signal-to-noise ratio (PSNR), structural similarity index (SSIM), mean squared error (MSE), learned perceptual image patch similarity (LPIPS), and natural image quality evaluator (NIQE) were recorded for each model. Since this study utilized an unpaired dataset, namely the R-F-datasetv2 railway fastening dataset, natural-light images with a similar structural enhancement result were chosen as comparative inputs, with NIQE values serving as the core indicator reference. This selection enables a more objective assessment of the model’s performance, ensuring that the enhancement results are comparable and reliable in practical applications. The specific quantitative comparison experiment results on the R-F-datasetv2 dataset are presented in Table 4. In these tables, an upward arrow (↑) indicates an improvement in value compared to the previous module addition, while a downward arrow (↓) indicates a decrease in value. Specifically, higher values of PSNR and SSIM denote better-enhanced image quality, whereas lower values of MSE, LPIPS, and NIQE denote better-enhanced image quality.

Based on the experimental results in Table 4, on the R-F-datasetv2 railway fastening dataset, the evaluation metrics PSNR, SSIM, MSE, LPIPS, and NIQE achieved by the CES-GAN algorithm are 18.435, 0.761, 1882.507, 0.119, and 2.597, respectively. All five evaluation metrics of our method are superior to the original EnlightenGAN enhancement algorithm. Specifically, the PSNR is 1.079 higher than the second-best method, EnlightenGAN, the SSIM is 0.069 higher than EnlightenGAN, the MSE is 632.88 lower than KinD, the LPIPS is 0.094 lower than Retinex-Net, and the NIQE is 0.196 lower than EnlightenGAN.

These results clearly demonstrate the superiority of the CES-GAN algorithm in image enhancement tasks. It effectively improves the quality of railway fastening images by increasing metrics such as peak signal-to-noise ratio (PSNR) and structural similarity (SSIM), while reducing the mean squared error (MSE). Additionally, significant improvements are observed in perceptual information loss (LPIPS) and natural image quality evaluation (NIQE) metrics.

#### 4.1.5. Qualitative Visualization Experiment Results and Analysis

The objective of this section is to evaluate the effectiveness of the CES-GAN method in enhancing low-light railway fastening images and to compare it with current low-light image enhancement methods. The R-F-datasetv2 dataset is chosen as the test benchmark to comprehensively assess the performance of each method in real-world scenarios. The following will present the comparative experimental results between the CES-GAN method and other low-light image enhancement methods and discuss their performance.

In this experiment, the CES-GAN method and other common low-light image enhancement methods were applied to the low-light railway fastening images in the R-F-datasetv2 dataset. These methods include LIME [35], MELLEN [36], URetinex [37], Retinex [38], EnlightenGAN [39], Zero-DCE [16], Self-DACE-stage1 [40], Self-DACE-stage2 [40], and KinD [41]. These methods are widely used in the field of image enhancement and serve as benchmarks for comparative analysis with CES-GAN to evaluate its strengths and weaknesses.

**Figure 7:**
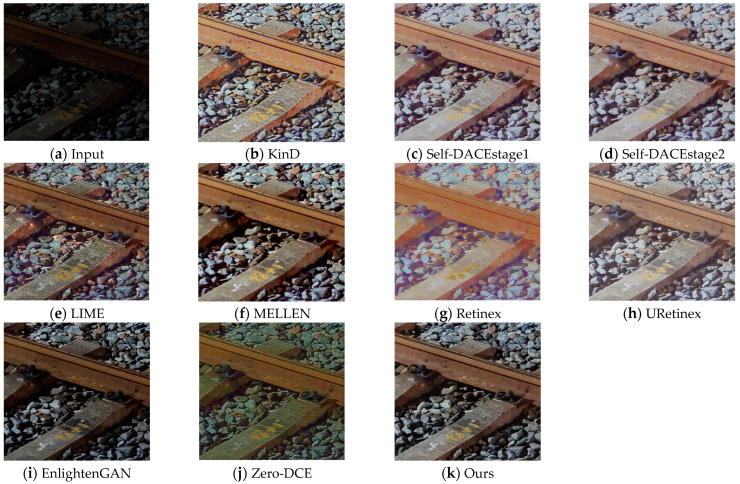
The visual comparison results of different low-light enhancement methods on the R-F-datasetv2 dataset.

**Figure 8:**
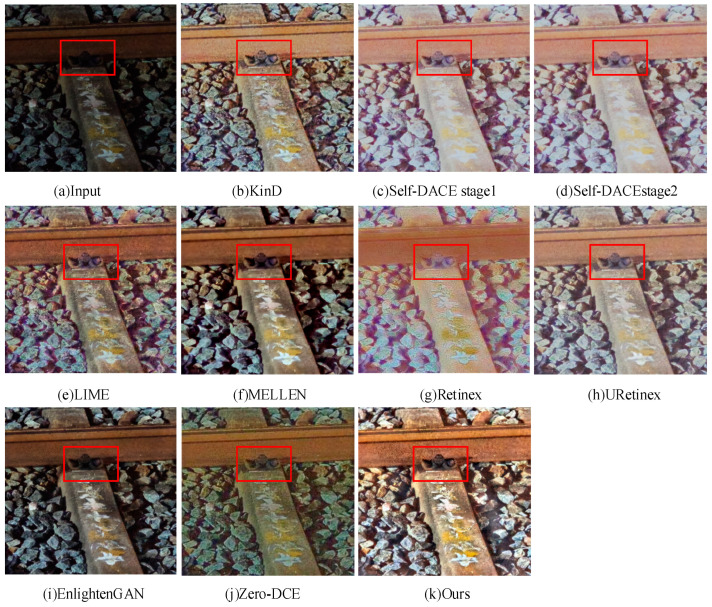
Visualization of different low-light enhancement methods on the R-F-datasetv2 dataset.

**Figure 9:**
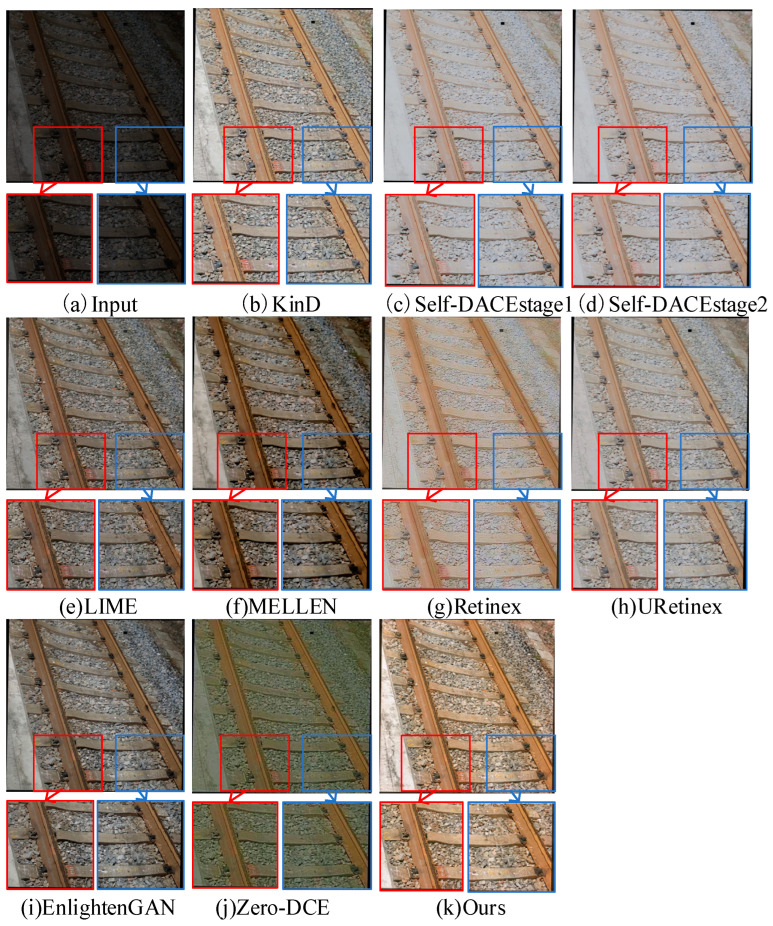
Visual comparison of local experimental results of different low-light enhancement methods on the R-F-datasetv2 dataset.

By conducting experiments on the R-F-datasetv2 dataset, this study allows for a visual observation of the enhancement effects of various methods on low-light railway fastening images. Specifically, the same images from the dataset are input into each model, and their output results are analyzed. Figure 7, Figure 8 and Figure 9 illustrate the comparative experimental results of various low-light image enhancement methods on the R-F-datasetv2 dataset.

Based on the observations from Figure 7, on the R-F-datasetv2 railway fastening dataset, several low-light image enhancement methods exhibit different limitations. Specifically, the LIME, EnlightenGAN, and Zero-DCE methods show issues of low brightness and detail loss in their enhancement results. The MELLEN method demonstrates excessive contrast and color distortion. Meanwhile, the enhancement results of the URetinex, Retinex, Self-DACE-stage1, and Self-DACE-stage2 methods exhibit problems of halos and color distortion, while the enhancement results of the KinD method show characteristics of blurred edge information.

In contrast, the enhancement method proposed in this paper demonstrates significant advantages on the R-F-datasetv2 dataset. Through observation, the enhancement results of our method maintain a moderate brightness while preserving the clarity of image details, and the colors are more consistent with the visual effect of natural images. This indicates that our method better meets the requirements of visual perception when handling low-light images of railway fastenings, thus exhibiting a higher enhancement effectiveness and image quality improvement capability compared to other methods.

Through the analysis of the results of low-light image enhancement methods on the R-F-datasetv2 dataset shown in Figure 8, the comparative results focus on the fastener in the railway. Additionally, the red box in Figure 8 represents a close-up of the details of the railway fasteners. By comparing the fastener-enhanced results, some noteworthy observations were made. Specifically, URetinex, Retinex, Self-DACE-stage1, and Self-DACE-stage2 methods exhibit color distortion in the fastener areas. Additionally, the image clarity is lower in the Retinex method, resulting in blurry fastener edge information. Furthermore, the MELLEN and EnlightenGAN methods produce railway fastener images with a darker brightness, while the KinD and LIME methods fade out detail information in the fastener area; for instance, the fastener grease location appears pale gray, which does not match the real scenario.

In contrast, the method proposed in this paper demonstrates significant advantages in enhancing railway fastener images. The fastener images enhanced by this method exhibit a brightness similar to reality, uniform color distribution, and well-preserved detail information. Compared to other methods, there is no apparent color distortion in the fastener area with this method, and both image clarity and edge information are well maintained.

To comprehensively evaluate the effectiveness of the proposed railway fastener low-light image enhancement method, this paper conducted in-depth testing on the R-F-datasetv2 railway fastener dataset and compared the results with commonly used low-light image enhancement methods. Specifically, the same images were inputted into each model to obtain low-light image enhancement results from various methods. Then, specific regions prone to enhancement issues, such as the lower left and lower right areas of the images, were separately magnified to compare the details of the enhancement results obtained on the R-F-datasetv2 railway fastener dataset. Figure 9 presents enlarged details of the enhanced images.

From the two enlarged areas in Figure 9, it can be observed that there are some issues with the enhancement results of various methods. For example, the enhancement result of the Zero-DCE method exhibits an overall greenish tint in the background, indicating a noticeable color bias. The enhancement result of the Retinex method shows color degradation and blurred edge information in the area of the fasteners. In the case of the LIME method, there is a grayish fastener in the bottom left corner with a significant color deviation. The MELLEN method’s enhancement result exhibits unclear contour shapes for the fasteners in the bottom right corner, with noticeable contour detail issues, and the overall image appears darker in color after enhancement. Similarly, the enhancement result of the EnlightenGAN method suffers from blurry contours and insufficient brightness in the fasteners in the bottom right corner. For the Self-DACE method, the enhanced fastener images in the bottom left and right corners exhibit texture loss and overexposure issues, with noticeable color differences compared to normal fasteners. In the case of the KinD and URetinex methods, the enhanced fasteners in the bottom left and right corners exhibit insufficient color saturation. The red box in Figure 9 represents a close-up of the railway fasteners in the lower left corner, and the blue box represents a close-up of the fasteners in the lower right corner. These close-ups are intended to better showcase the texture details of the enhancement results, allowing for a more thorough evaluation of each method’s performance.

In contrast, the enhancement results of the proposed method show that the fasteners in the bottom left and right corners have been effectively lifted from the low-light regions. The fasteners and their surrounding textures are clearer, with moderate brightness and no issues of overexposure or color distortion. This indicates a significant advantage of our method in the task of enhancing low-light railway fastener images, as it can effectively improve image quality and enhance the presentation of fastener details.

**Figure 10:**
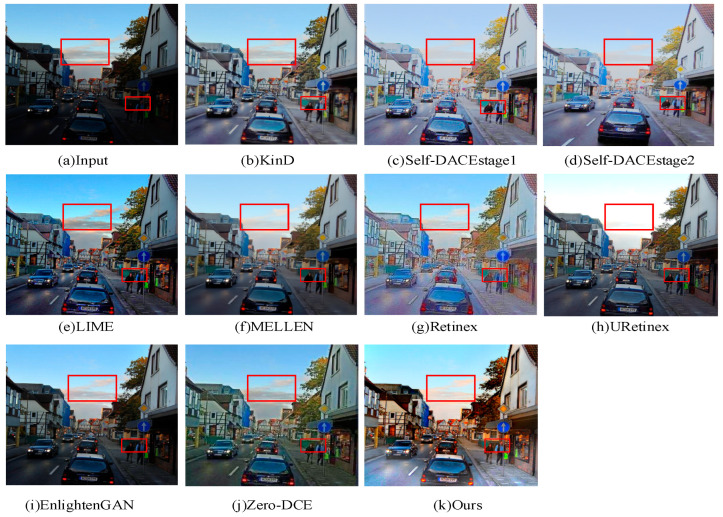
The visual comparison results of different low-light enhancement methods on the LOL dataset.

To further validate the generalization performance of our proposed method, we randomly selected an image from the LOL dataset for qualitative analysis. The specific results are presented in Figure 10.

According to the results presented in Figure 10, the enhancement result of the Self-DACE method exhibits weak contrast in the clouds in the sky, with blurry contours and pale colors. In contrast, the URetinex method directly loses many details of the clouds, making their contours difficult to discern. In the Retinex method, significant noise and artifacts are present, and the basic contours of the faces are difficult to recognize. The images enhanced by the LIME and EnlightenGAN methods overall exhibit a darker tone, with some areas still appearing extremely dark. Meanwhile, the images enhanced by the Zero-DCE and KinD methods have blurry overall contours, and the basic contours of the people are not clearly displayed. In comparison, the images enhanced by our proposed method have moderate brightness, relatively clear textures in the basic contours of the faces, restored colors in the sky and clouds, and no overexposure in other areas. The texture details and contours are preserved relatively intact, further demonstrating the generalization performance of our method.

## 5. Conclusions

In this paper, we proposed an unsupervised low-light enhancement method called CES-GAN based on contrastive learning GAN. In terms of network model design, we provided a detailed description of the overall network architecture, with a particular focus on the design of the generator and discriminator. In the generator structure, we introduced a composite loss function consisting of feature consistency loss, contrastive learning loss, and illumination loss to assist the generator in learning the distribution characteristics of the data. Particularly, the introduction of contrastive learning loss helps to improve the generalization ability of the generator and enables it to learn subtle features of the data. In the discriminator structure, we employed both global and local discriminators, the combination of which aids in the model’s comprehensive understanding of the content and structure of the images.

The experimental part thoroughly validated and analyzed the proposed method in this paper. Regarding the experimental setup, detailed descriptions of the datasets and evaluation metrics used in the experiments were provided. Subsequently, the paper conducted ablation experiments and analyses, comparing the performance differences among different models to validate the effectiveness of CES-GAN. In the quantitative experimental results and analysis, multiple evaluation metrics were employed to comprehensively evaluate the model’s performance and compare it with other classical methods, further confirming the superiority of CES-GAN. Finally, in the qualitative visualization experimental results and analysis, the enhancement effects of CES-GAN in different scenarios were demonstrated through intuitive visualizations. The results were deeply analyzed and discussed, proving the effectiveness and performance advantages of the proposed method.

## Figures and Tables

**Table 1:** Equipment and parameters used in the experiment.

Equipment	Parameters	Equipment	Parameters
GPU	NVIDIA TITAN V 12G	Cuda	11.1
CPU	Intel(R) Xeon(R) Silver4114 CPU	Framework	pytorch
RAM	400 GB		
OS	Linux		

**Table 2:** Ablation quantitative results of the CES-GAN algorithm on the R-F-datasetv2 dataset.

Method	PSNR↑	SSIM↑	MSE↓	LPIPS↓	NIQE↓
Semantic Information + Contrastive Learning	16.758	0.716	2461.668	0.257	2.981
Contrastive Learning + Illumination Consistency	17.775	0.743	2172.236	0.173	2.797
Semantic Information + Illumination Learning	17.703	0.729	2339.287	0.209	2.851
Semantic Information + Contrastive Learning + Illumination Consistency	18.456	0.781	1882.507	0.119	2.597

**Table 3:** Ablation quantitative results of the CES-GAN algorithm on the LOL dataset.

Method	PSNR↑	SSIM↑	MSE↑	LPIPS↓	NIQE↓
Semantic Information + Contrastive Learning	16.246	0.698	2564.565	0.293	2.985
Contrastive Learning + Illumination Consistency	17.066	0.725	2439.287	0.233	2.846
Semantic Information + Illumination Learning	16.847	0.718	2434.923	0.274	2.902
Semantic Information + Contrastive Learning + Illumination Consistency	17.783	0.746	2193.696	0.185	2.717

**Table 4:** Quantitative comparison experiment results on the R-F-datasetv2 railway fastening dataset.

Dataset	Method	PSNR↑	SSIM↑	MSE↓	LPIPS↓	NIQE↓
	LIME [35]	12.718	0.318	3477.896	0.709	2.622
	MBLLEN [36]	11.885	0.398	4212.931	0.712	4.385
	URetinex [37]	11.977	0.371	4124.537	0.705	3.219
	Retinex [38]	12.330	0.377	3803.010	0.668	3.756
	Zero-DCE [16]	16.758	0.594	4497.388	0.455	3.533
R-F-datasetv2	EnlightenGAN [39]	17.356	0.692	2515.387	0.213	3.011
	Self-DACE-stage1 [40]	11.914	0.479	4184.778	0.710	3.532
	Self-DACE-stage2 [40]	11.926	0.489	4193.250	0.709	4.160
	KinD [41]	13.003	0.368	3256.706	0.676	3.208
	Ours	18.435	0.761	1882.507	0.119	2.597

## Data Availability

The data used to support the findings of this study are available from the author upon request.
